# *CMAS* and *ST3GAL4* Play an Important Role in the Adsorption of Influenza Virus by Affecting the Synthesis of Sialic Acid Receptors

**DOI:** 10.3390/ijms22116081

**Published:** 2021-06-04

**Authors:** Yaxin Zhao, Jiahui Zou, Qingxia Gao, Shengsong Xie, Jiyue Cao, Hongbo Zhou

**Affiliations:** 1 State Key Laboratory of Agricultural Microbiology, College of Veterinary Medicine, Huazhong Agricultural University, Wuhan 430070, China; YaxinZhao@webmail.hzau.edu.cn (Y.Z.); zoujiahui@webmail.hzau.edu.cn (J.Z.); gaoqingxia@163.com (Q.G.); 2 College of Veterinary Medicine, Huazhong Agricultural University, Wuhan 430070, China; ssxie@mail.hzau.edu.cn

**Keywords:** *CMAS*, *ST3GAL4*, CRISPR/Cas9, influenza, sialic acid receptors

## Abstract

Influenza A viruses (IAVs) initiate infection by attaching Hemagglutinin (HA) on the viral envelope to sialic acid (SA) receptors on the cell surface. Importantly, HA of human IAVs has a higher affinity for α-2,6-linked SA receptors, and avian strains prefer α-2,3-linked SA receptors, whereas swine strains have a strong affinity for both SA receptors. Host gene *CMAS* and *ST3GAL4* were found to be essential for IAV attachment and entry. Loss of *CMAS* and *ST3GAL4* hindered the synthesis of sialic acid receptors, which in turn prevented the adsorption of IAV. Further, the knockout of *CMAS* had an effect on the adsorption of swine, avian and human IAVs. However, *ST3GAL4* knockout prevented the adsorption of swine and avian IAV and the impact on avian IAV was more distinct, whereas it had no effect on the adsorption of human IAV. Collectively, our findings demonstrate that knocking out *CMAS* and *ST3GAL4* negatively regulated IAV replication by inhibiting the synthesis of SA receptors, which also provides new insights into the production of gene-edited animals in the future.

## 1. Introduction

Influenza A virus (IAV) is an upper respiratory pathogen in humans and animals, and it evolves rapidly and can lead to seasonal epidemics and occasional pandemics [1,2]. As IAVs are highly prone to mutation and newly transmitted viruses can quickly acquire drug resistance, the efficacy of current vaccines and drugs has been greatly reduced [3,4]. Therefore, the need to develop new host-directed therapeutics against IAVs is urgent [5,6,7].

This study focuses on the host gene *CMAS* and *ST3GAL4*, which were included in the previous genome-wide CRISPR knockout screening. Cytidine monophosphate N-acetylneuraminic acid synthetase (*CMAS*), also called CMP-Sialic acid synthetases, catalyzes the conversion of N-acetylneuraminic acid (NeuNAc) to cytidine 5’-monophosphate N-acetylneuraminic acid (CMP-NeuNAc), which is an essential intermediate for the synthesis of sialic acid receptors [8]. ST3 beta-galactoside alpha-2,3-sialyltransferase 4 (*ST3GAL4*), a member of the sialyltransferase family, also participated in the synthesis of sialic acid receptors [9]. Sialic acid receptor synthesis is a large and complex network involving dozens of enzymes and chemicals [10]. The biosynthesis of sialylated oligosaccharide sequences depends on the catalysis of the sialyltransferase family, and these enzymes transfer the sialic acid group in the active intermediate product, CMP-sialic acid, to the glycoprotein and terminal position on the sugar chain in different ways [8,11,12]. They are divided into different families (ST6Gal, ST6GalNAc, ST3Gal and ST8Sia) according to the types of glycosidic bonds newly formed after the transfer, and the ST3 family transfers sialic acid groups to galactose residues mainly based on α-2,3 glycosidic bond [11].

The life cycle of IAV can be roughly divided into five stages: adsorption, invasion, replication, assembly and budding [13,14,15]. Interference or interruption of any stage will cause the replication of IAVs to fail. The HA of IAVs can bind to SA receptors on the surface of host cells, which is the initial step of IAVs infection [16,17]. Different species of IAVs have different preferences for different types of sialic acid receptors. For instance, HA of human IAVs has a higher affinity for receptors with α-2,6-linked sialic acid, whereas horse and avian strains prefer α-2,3-linked sialic acid [18,19]. The proportions of α-2,3-linked and α-2,6-linked sialic acid receptors on the surface of pig cells are equivalent, so swine strains are more likely to have a strong affinity for both sialic acid receptors at the same time [4]. Hence, IAVs reassorted in pigs were capable of spreading across species [20].

To determine the relationships of IAV and these two genes, we established the *CMAS* and *ST3GAL4* knockout newborn pig tracheaepithelial (NPTr) cells by using the CRISPR/Cas9 system and found that knocking out *CMAS* and *ST3GAL4* had an effect on the replication of H1N1 strains. These two genes were further verified to play an important role in the formation of sialic acid receptors. The knockout of *CMAS* impaired the synthesis of α-2,3-linked and α-2,6-linked sialic acid receptors, which led to a significant inhibitory effect on the adsorption of IAVs, and ultimately inhibited the proliferation of swine, avian and human IAVs. Besides, knocking out *ST3GAL4* hindered the synthesis of α-2,3-linked sialic acid receptors, and had a momentous inhibitory impact on the proliferation of swine and avian IAVs, but had little effect on the replication of human IAVs.

Taken together, we described the distinct role of the *CMAS* and *ST3GAL4* for the synthesis of sialic acid receptors. When the host cell lacks *CMAS* or *ST3GAL4*, the synthesis of acid receptors on its surface was hindered, which led to different adsorption inhibition of IAVs that recognize α-2,3-linked or α-2,6-linked sialic acid receptors.

## 2. Results

### 2.1. Generation of Gene Knockout Cell Lines

**Figure 1:**
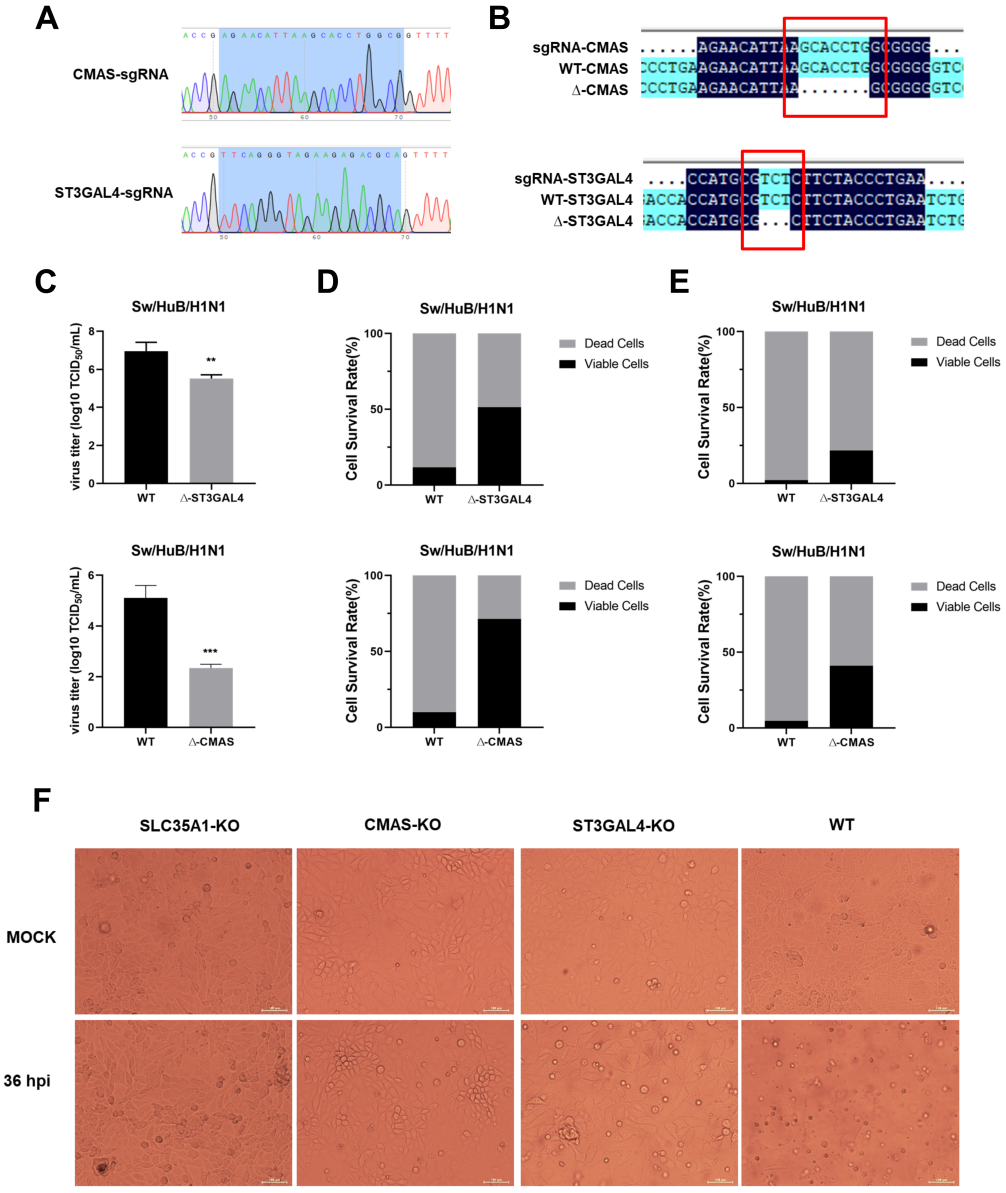
Generation of knockout cell line. (**A**) sgRNA sequencing results. (**B**) Detection of sgRNA knockout efficiency. (**C**) Effect of gene knockout on IAV replication. *CMAS*-KO, *ST3GAL4*-KO and *SLC35A1*-KO NPTr cells were generated through the CRISPR/Cas9 system; NPTR-Cas9 NPTr cells were used as wild-type control. WT NPTr cells and KO NPTr cells were infected with Sw/HuB/H1N1 virus (MOI = 0.01). Cell supernatants were collected at 36 hpi, and the virus titers were determined by TCID_50_ assay on MDCK cells. (Data from three independent experiments, mean ± SD; ** *p* < 0.01; *** *p* < 0.001; two-tailed Student’s *t* test.) (**D**) MOI = 0.01 or (**E**) MOI = 0.1, WT NPTr cells and KO NPTr cells were infected or not infected with Sw/HuB/H1N1 virus, and the cell survival rate was determined by calculating the ratio of cells number in the infected group divided by the cells number in the uninfected group. (**F**) The survival of each monoclonal cell line at 36 h post-infection with or without Sw/HuB/H1N1 virus. Scale bar = 100 µM.

Newborn pig tracheaepithelial (NPTr) cells stably expressing Cas9 protein were used to construct knockout cell lines. The *CMAS*-sgRNA or *ST3GAL4*-sgRNA were cloned in a LentiGuide-Puro vector and confirmed by sequencing, as shown in Figure 1A. After infection with lentivirus, the polyclonal knockout cell line was obtained. After extraction of the genome, the PCR amplification products were connected to the T vector and transformed, and the single-colony sequencing was carried out. Seven (*CMAS*) or three (*ST3GAL4*) bases were found missing where sgRNA was located, compared with the original sequence, which verified the effective knockout of polyclonal cell lines (Figure 1B). The virus titer was determined at 36 h post-infection (hpi), and the cell-survival rates under different multiplicities of infection (MOI) conditions were calculated at 72 hpi. The results showed that knockout of *CMAS* or *ST3GAL4* could reduce virus titer and increase the survival rates of cells under different MOI, compared with WT NPTr cells (Figure 1E).

Subsequently, *CMAS* knockout efficiency was detected by Western blotting (Figure S1A). However, due to the lack of specific antibodies against swine *ST3GAL4*, we could not conduct further verification of the knockout efficiency of monoclonal cell lines by Western blotting. The *ST3GAL4*-KO-2 cell line was determined to possess the best antiviral effect by measuring the virus titer and cell survival rate at 36 h after infection with the Sw/HuB/H1N1 strain, so it was selected for the following work (Figure S1B).

It has been reported that knocking out *SLC35A1* had a great inhibitory effect on the replication of IAV because it simultaneously interfered with the synthesis of α-2,3-linked and α-2,6-linked sialic acid receptors, so we used *SLC35A1*-KO NPTr cells as a positive control. For each cell line, we also set up a set of blank controls (MOCK) that were not infected with a virus. At 36 hpi, we observed the survival of KO NPTr cells. It was found that almost all WT NPTr cells died at 36 hpi, while both *CMAS*-KO and *ST3GAL4*-KO NPTr cells survived, and the survival number of *CMAS*-KO NPTr cells was more. For this, we preliminarily determined the inhibitory effect of knocking out *CMAS* and *ST3GAL4* on the Sw/HuB/H1N1 strain (Figure 1F).

### 2.2. Knockout of CMAS or ST3GAL4 Has No Effect on Cell Viability

**Figure 2:**
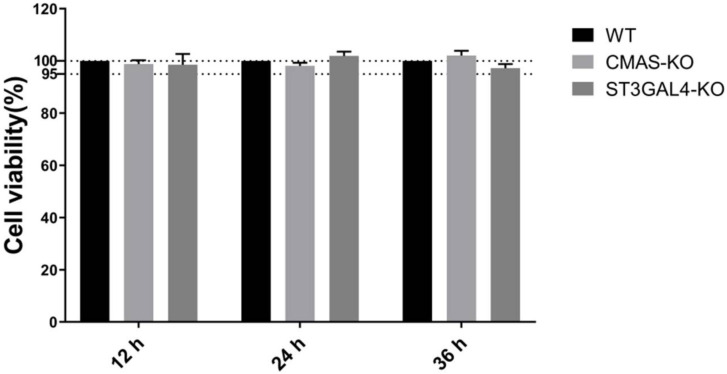
Knockout of *CMAS* or *ST3GAL4* has no effect on cell viability. *CMAS*-KO and *ST3GAL4*-KO NPTr cells were generated through the CRISPR/Cas9 system; NPTR-Cas9 cells were used as wild-type control. WT NPTr cells and KO NPTr cells were seeded in 96-well plates and the cell viability was measured at 12, 24 and 36 h.

A CCK-8 kit was used for detection in order to determine whether the *CMAS* or *ST3GAL4* gene knockout had an effect on cell viability. The results showed that the viability of the knockout cells was not significantly different from that of the control cells. Therefore, the knockout of *CMAS* and *ST3GAL4* had a bare effect on the normal growth of cells (Figure 2).

### 2.3. Knockout of CMAS or ST3GAL4 Negatively Modulates the Proliferation of Swine IAV

**Figure 3:**
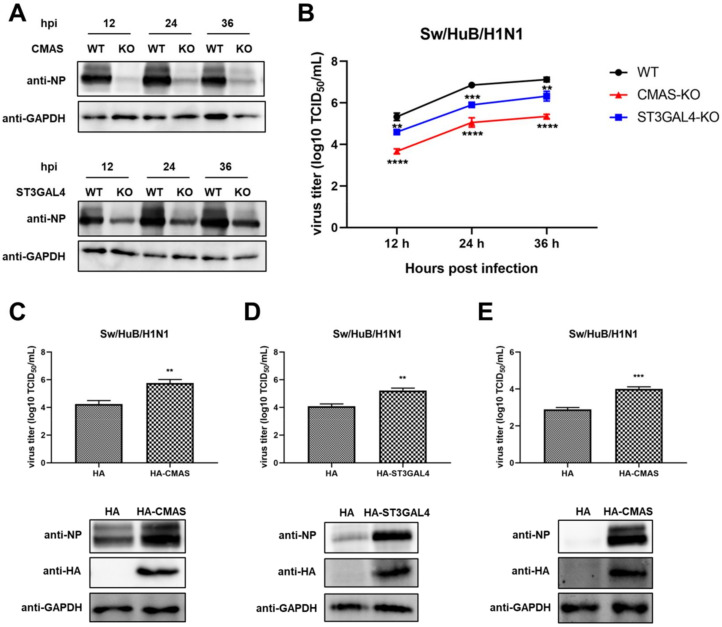
Knockout of *CMAS* or *ST3GAL4* negatively modulates the proliferation of the swine influenza virus. *CMAS*-KO and *ST3GAL4*-KO NPTr cells were generated through the CRISPR/Cas9 system. NPTR-Cas9 cells were used as wild-type control. (**A**,**B**) Effects of KO cell lines on the proliferation of swine influenza viruses. WT NPTr cells and KO NPTr cells were infected with Sw/HuB/H1N1 virus (MOI = 0.01); cell supernatants and lysates were collected at 12, 24 and 36 hpi. (**A**) Viral protein expressions were detected by Western blotting or (**B**) virus titers were determined by TCID_50_ assay on MDCK cells. (Data from three independent experiments, mean ± SD; ** *p* < 0.01; *** *p* < 0.001; **** *p* < 0.0001; one-way ANOVA.) (**C**,**D**) pCAGGS-HA (HA), HA-*CMAS* or HA-*ST3GAL4* plasmid was transfected into WT NPTr cells, and inoculated with a Sw/HuB/H1N1 virus 24 h later (MOI = 0.01); then, cell supernatants and lysates were collected at 24 hpi and virus titers were determined by TCID_50_ assay on MDCK cells or viral proteins expression were detected by Western blotting. (**E**) pCAGGS-HA (HA) or HA-*CMAS* plasmid was transfected into *CMAS*-KO NPTr cells, and inoculated with a Sw/HuB/H1N1 virus 24 h later (MOI = 0.01); then, cell supernatants and lysates were collected at 24 hpi and virus titers were determined by TCID_50_ assay on MDCK cells or viral proteins expression were detected by Western blotting. (Data from three independent experiments, mean ± SD; ** *p* < 0.01; *** *p* < 0.001; two-tailed Student’s *t* test.).

To further explore the relationship between IAV replication and gene knockout, a Western blot assay was performed to detect the expression of viral nucleoprotein (NP) at different time points after virus infection in both WT cells and KO cells. The results showed that the NP expression level of the *CMAS* knockout cell line was significantly decreased compared with WT cells, while the NP expression level of the *ST3GAL4* knockout cell line was also decreased, but the decline is not as obvious as that of the *CMAS* knockout cell line (Figure 3A). To confirm this result, a TCID_50_ assay was performed after collecting the supernatants of each cell line at different time points after virus infection (Figure 3B). The results are consistent with cell survival and NP expression results and showed that knockout *CMAS* or *ST3GAL4* had a significant inhibitory effect on the proliferation of swine IAV and the former had a greater effect.

Since knocking out these two genes will inhibit the replication of swine IAV, overexpression of these two genes is likely to promote the growth of swine IAV. To this end, the HA-*CMAS* or HA-*ST3GAL4* plasmids were transfected in WT NPTr cells, and then the WT NPTr cells were infected with Sw/HuB/H1N1 after 24 h post-transfection. The cell supernatants and lysates were collected at 24 hpi. The results showed that overexpression of *CMAS* or *ST3GAL4* could indeed cause the enhancement of swine IAV replication (Figure 3C,D). At the same time, we also overexpressed the HA-*CMAS* plasmid in *CMAS*-KO cells, and the results indicated that the replication ability of IAV could indeed be increased when the gene function was restored (Figure 3E).

### 2.4. Effects of Gene Knockout on the Proliferation of IAVs of Different Species

**Figure 4:**
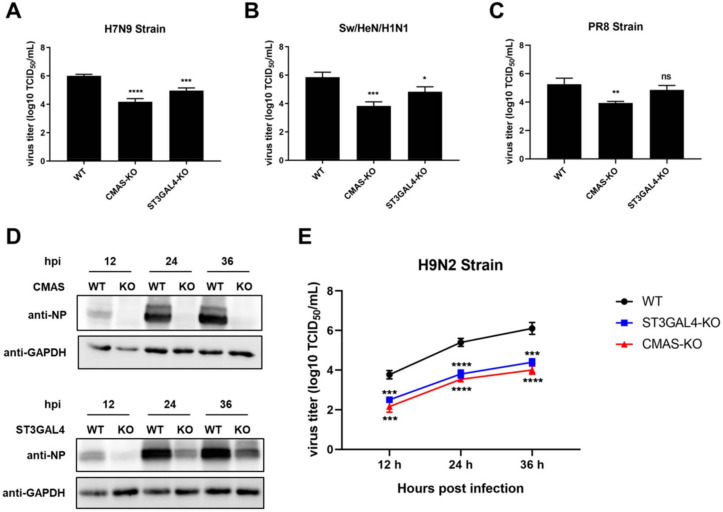
Effects of KO cell lines on the proliferation of influenza viruses of different species. *CMAS*-KO and *ST3GAL4*-KO NPTr cells were generated through the CRISPR/Cas9 system; NPTR-Cas9 cells were used as wild-type control. (**A**–**C**) Effects of KO cell lines on the proliferation of influenza viruses of different species at 36 hpi. WT NPTr cells and KO NPTr cells were infected with (**A**) H7N9 virus, (**B**) Sw/HeN/H1N1 virus or (**C**) PR8 virus (MOI = 0.01). Cell supernatants were collected at 36 hpi, and virus titers were determined by TCID_50_ assay on MDCK cells. (Data from three independent experiments, mean ± SD; * *p* < 0.05; ** *p* < 0.01; *** *p* < 0.001; one-way ANOVA). (**D**,**E**) Effects of KO cell lines on the proliferation of H9N2 influenza viruses. WT NPTr cells and KO NPTr cells were infected with the H9N2 virus (MOI = 0.01). Cell supernatants and lysates were collected at 12, 24, and 36 hpi. (**D**) Viral protein expressions were detected by Western blotting or (**E**) virus titers were determined by TCID_50_ assay on MDCK cells. (Data from three independent experiments, mean ± SD; * *p* < 0.05; ** *p* < 0.01; *** *p* < 0.001; **** *p* < 0.0001; ns represented no significance; one-way ANOVA).

To confirm the inhibition of virus replication by knocking out *CMAS* and *ST3GAL4* is not strain-specific, we used a variety of different IAV subtypes, including swine strain Sw/HeN/H1N1 that recognized α-2,3 and α-2,6 sialic acid receptors, avian strain H7N9 that mainly recognized α-2,3 receptors and PR8 strain isolated from human that mainly recognized α-2,6 receptors, to carry out TCID_50_ assay. The results of virus titer at 36 hpi showed that the knockout of *CMAS* inhibited the replication of all the above strains, and knockout of *ST3GAL4* was effective against swine and avian influenza strains, but not against human influenza strains (Figure 4A–C).

It has been reported that *ST3GAL4* played a role in the synthesis of α-2,3 sialic acid receptors, which may cause its knockout to have a more significant antiviral effect on avian strains. Besides, there are no detailed reports on how *CMAS* affects the synthesis of different types of sialic acid receptors, but because it plays a role in the upstream of the sialic acid pathway, we speculate that *CMAS* has an effect on the synthesis of α-2,3 and α-2,6 sialic acid receptors. Therefore, we compared the effects of *CMAS* or *ST3GAL4* knockout on avian influenza and swine influenza. Therefore, we infected WT cells and KO cells with the avian H9N2 strain, and found that the inhibition of *ST3GAL4* knockout on the proliferation of H9N2 avian IAV was stronger than on swine IAV (Figure 4D,E).

### 2.5. The Effect of CMAS and ST3GAL4 on the Synthesis of Different Types of Sialic Acid Receptors

On the basis of existing related research, we understand that *CMAS* and *ST3GAL4* were involved in the formation of different types of sialic acid receptors, but we still need to verify this function further. To this end, we launched a sialic lectin analysis experiment.

**Figure 5:**
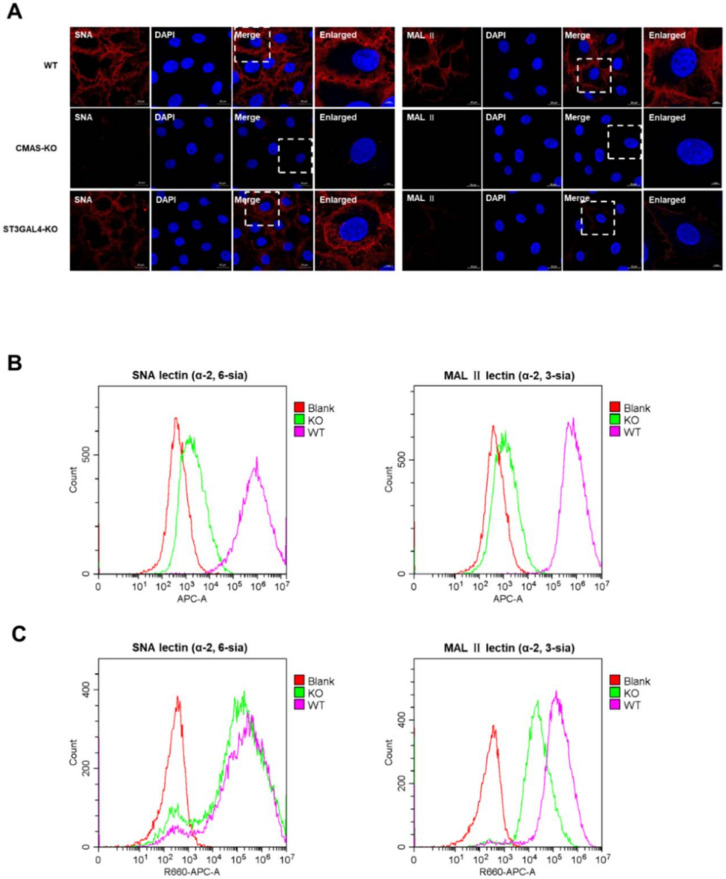
The effect of CMAS and ST3GAL4 on the synthesis of different types of sialic acid receptors. CMAS-KO and ST3GAL4-KO NPTr cells were generated through the CRISPR/Cas9 system; NPTR-Cas9 cells were used as wild-type control. (A–C) The expression of sialic acid was analyzed by lectin staining. WT NPTr cells and KO NPTr cells were treated with lectins specific for α-2,6-linked sialic acid (SNA) or α-2,3-linked sialic acid (MAL). (A) Fluorescent microscopy analysis of the effect of CMAS or ST3GAL4 knockout on the synthesis of sialic acid receptors. (B) Flow cytometry analysis of the effect of CMAS knockout on the synthesis of sialic acid receptors. (C) Flow cytometry analysis of the effect of ST3GAL4 knockout on the synthesis of sialic acid receptors. Blank cells were stained with only Cy5-streptavidin, but not treated with lectin. Scale bar = 20 μm or 5 μm.

It was reported that Maackia amurensis lectin II [MAL II] could specifically combine with the α-2,3-linked sialic acid receptor, and Sambucus nigra lectin (SNA) had more preference in combination with α-2,6-linked sialic acid receptor [21]. Both biotinylated lectins were purchased from Vector Biolabs, and were incubated to mark the sialic acid cell surface receptors, and then stained with streptavidin conjugated to Cy5. Flow cytometry and confocal microscopy analysis of WT cells and KO cells showed the deletion of *CMAS* had a significant effect on the synthesis of both types of sialic acid receptors (Figure 5A,B), and the decrease of *ST3GAL4* had a great effect only on the synthesis of α-2,3-linked sialic acid receptors, but had little effect on the synthesis of α-2,6-linked sialic acid receptors (Figure 5A,C), which revealed the limitation at one key stage in the life cycle of the IAV after knocking out these two genes.

### 2.6. CMAS and ST3GAL4 Are Essential for IAVs Attachment

**Figure 6:**
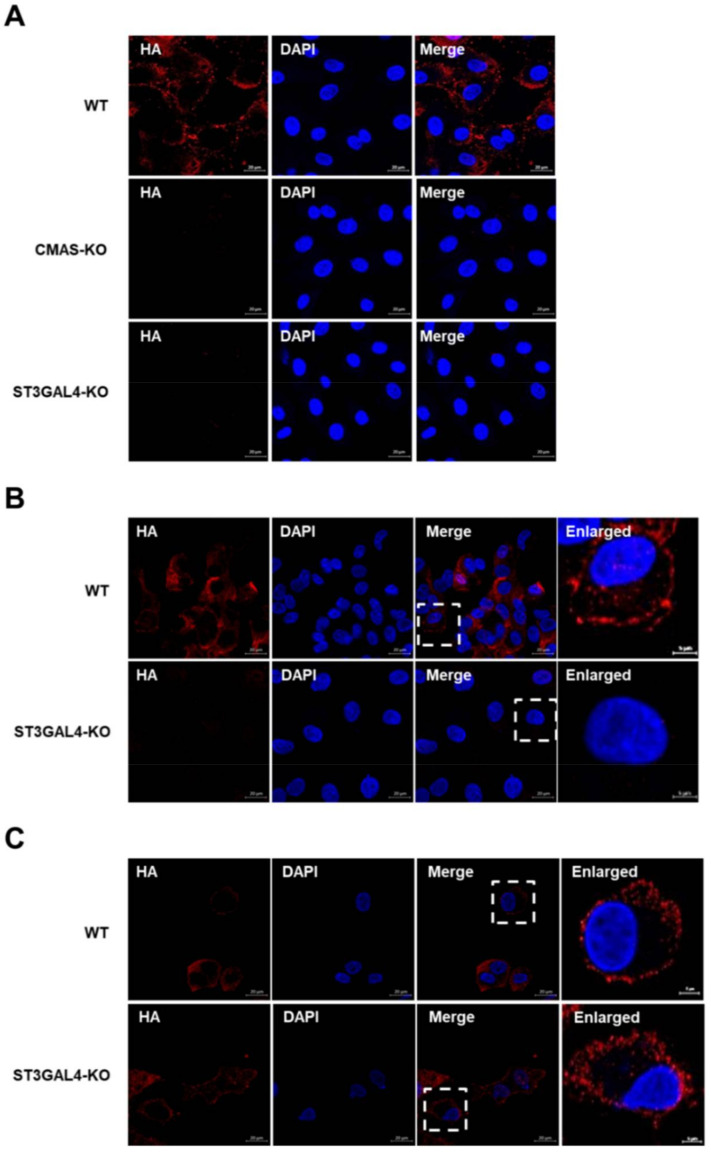
*CMAS* and *ST3GAL4* are essential for IAVs attachment. *CMAS*-KO and *ST3GAL4*-KO NPTr cells were generated through the CRISPR/Cas9 system; NPTR-Cas9 cells were used as wild-type control. (**A**) WT NPTr cells and KO NPTr cells were infected with Sw/HuB/H1N1 virus (MOI = 75) for 1 on ice and incubated with influenza virus HA protein antibody, then analyzed by fluorescent microscopy. Scale bar = 20 μm. (**B**,**C**) The lack of *ST3GAL4* mainly prevents the avian influenza virus attachment. *ST3GAL4*-KO cells or control cells were infected with (**B**) H9N2 or (**C**) PR8 virus (MOI = 75) for 1 h on ice and analyzed by fluorescent microscopy. Scale bar = 20 μm or 5 μm.

Thus far, we have explained why the replication of different species of IAVs was inhibited in varying degrees by knocking out *CMAS* or *ST3GAL4* through lectin-analysis experiments. Since the lack of target genes impaired the synthesis of sialic acid receptors, and sialic acid receptors are crucial for IAV adsorption, the lack of *CMAS* or *ST3GAL4* may block the IAV attachment. The KO cells or control cells were infected with a Sw/HuB/H1N1 strain for 1 h on ice, which allowed attachment but not internalization. We found that the gene knockout cell lines had a significant effect on viral attachment compared with wild-type cells by detecting the anti-HA antibody (Figure 6A).

Since human IAVs mainly bind to α-2,6 sialic acid receptors and avian IAVs prefer to bind to α-2,3 sialic acid receptors, we compared the effect of *ST3GAL4* knockout on the attachment of avian and human IAVs. The results showed that the knockout of *ST3GAL4* had a significant effect on inhibiting the virus attachment of avian H9N2 subtype strains, while the effect on PR8 subtype strains was not too obvious (Figure 6B,C). This result was also consistent with our previous findings in virus titer and lectins analysis.

### 2.7. CMAS Interacts with IAV M1

**Figure 7:**
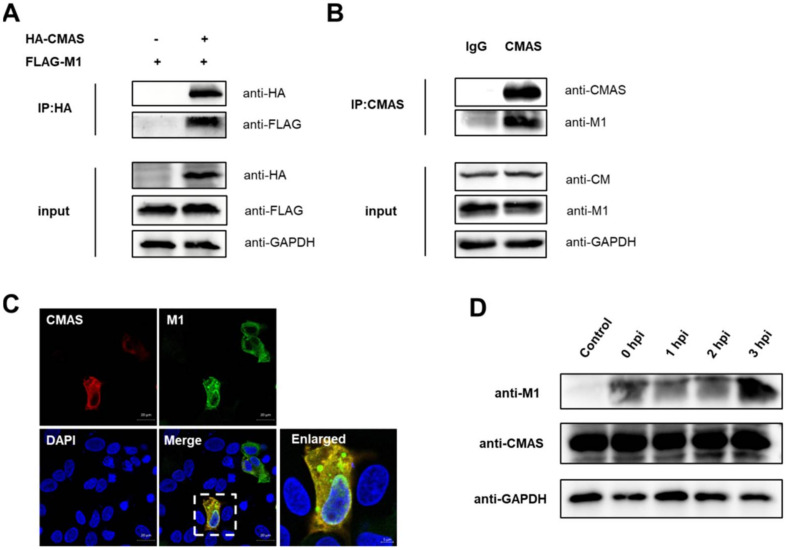
*CMAS* interacts with IAV M1. (**A**,**B**) Immunoblot analysis of the interactions between *CMAS* and M1. (**A**) HEK293T cells were transfected with HA-*CMAS* plasmid and Flag-M1 plasmid and followed by lysing at 24 h post-transfection. Co-IP assay was carried out using an anti-HA antibody. (**B**) The endogenous interaction between *CMAS* and M1. WT NPTr cells were infected with Sw/HuB/H1N1 strain and followed by lysing at 24 hpi. Immunoblot analysis was performed by using an anti-*CMAS* rabbit antibody or rabbit IgG. (**C**) Colocalization of *CMAS* and M1. WT NPTr cells were transfected with HA-*CMAS* and plasmid Flag-M1 plasmid then fixed at 24 h post-transfection. Colocalization of *CMAS* and M1 was analyzed by confocal microscopy. Scale bar = 20 μm. (**D**) WT NPTr cells were infected or not infected with Sw/HuB/H1N1 strain, and placed on ice for 1 h for IAVs adsorption to ensure that the life process of each group of IAVs was consistent. The cell lysates were collected at the designated time point for Western blot analysis.

Our experiments have proved that *CMAS* has a significant inhibition on the replication of IAV, so we hope to find its extra function besides affecting the adsorption process. We have successively explored the interaction between *CMAS* and the main subunits of IAV, and finally found that *CMAS* interacts with influenza virus matrix protein 1 (M1). By co-transfecting pCAGGS-HA-*CMAS* (HA-*CMAS*) and p3xflag-cmv14-M1 (FLAG-M1) plasmids on 293T cells, the interaction between the two was found (Figure 7A). The swine influenza Sw/HuB/H1N1 strain was used to infect wild-type NPTr cells. Cell lysates were collected 24 h after infection for endogenous testing. The results also showed that there is an interaction between the two (Figure 7B). In addition, the two plasmids were co-transfected on wild-type NPTr cells, and the colocalization of the two was found by laser confocal microscopy analysis (Figure 7C). M1 plays a role mainly in the early stage of the IAV life cycle (within 3 hpi). During this period of time, we tested the effect of IAV M1 protein on the level of CMAS and found that the levels of CMAS were not affected by M1 over time (Figure 7D). These results indicate that *CMAS* may also have a certain effect on the related virus life activities that M1 participates in, such as virus unpacking, nuclear export and budding, but the specific mechanism is still unknown.

## 3. Discussion

Differences in receptor-binding specificity of IAVs depend on host restriction, which creates a species barrier and makes some IAVs not capable of infecting other species [22,23]. As mentioned in the introduction section, human-origin IAVs have a higher affinity for receptors with α-2,6-linked sialic acid, and horse and avian strains preferentially recognize α-2,3-linked receptors, whereas swine IAVs have good binding ability to these two receptors. This is also the main research background of this study [20,24].

In this study, the host gene *CMAS* and *ST3GAL4* were selected as the research objects based on the results of whole-genome screening in our laboratory. The gene knockout NPTr cells were established by using the CRISPR/Cas9 system. For such cell lines, we preliminarily verified that it had an inhibitory effect on the proliferation of IAV. Moreover, the knockout of *CMAS* or *ST3GAL4* did not affect the cell viability compared with the wild-type cells.

Then, we found that *CMAS* and *ST3GAL4* interfered with the synthesis of sialic acid receptors, which hindered the binding of IAV to the sialic acid receptors on the cell surface, thus leading to the failure of subsequent stages of the IAV life cycle. This revealed the mechanism of these two genes’ knockout against IAV. It is worth noting that the knockout of *CMAS* affected the synthesis of α-2,3 and α-2,6 sialic acid receptors at the same time, and ultimately prevents the adsorption process of swine, avian and human IAVs.

However, knocking out *ST3GAL4* had an impact on the adsorption of avian and swine IAV but not human IAV, due to the fact that swine, avian and human IAVs had different preferences for different types of sialic acid receptors and *ST3GAL4* affected the synthesis of α-2,3 sialic acid receptors. As there were reports that swine IAV could also enter cells through both α-2,3 and α-2,6 sialic acid receptors, the knockout of *ST3GAL4* had a smaller effect on the proliferation of swine IAV, compared to avian IAV. This was demonstrated and verified in our previous TCID_50_ results and virus attachment assay experiments. It is worth noting that in the same batch of experiments, the NPTr cells infected or the non-infected control-sgRNA lentivirus had no significant impact on the proliferation of the virus. Therefore, it is also appropriate to use NPTr cells that stably expressed Cas9 as wild-type controls in our study (Figure S2).

*CMAS* was found to cause intellectual disability (ID) when it occurred with homozygous mutations in a previous study [25]. The discovery provides a new candidate gene, *CMAS*, for the clinical diagnosis of ID. The impact of *CMAS* on a viral infection has not been studied in detail so far, but *CMAS* was found in Han’s screen of IAV replication-related host genes and it ranked high, which is consistent with the previous screening results of our laboratory [21]. It is precisely because *CMAS* is of great significance to the replication of IAV in our research that we hope to find some of its functions in addition to affecting the adsorption process. Therefore, we discovered its interaction with IAV M1. However, since we cannot rule out the influence of *CMAS* restricting the virus adsorption on the consequent steps at present, we do not yet know its additional role in the replication of IAV, but this can also provide clues for further related work. Importantly, previous studies have suggested that sialic acid may not be the only receptor for IAVs to enter host cells [26,27]. Other receptor, such as Annexin V, can also serve as potential adsorption receptors for IAVs, so there are still some influenza viral particles that can enter cells through non-classical receptors or other mechanisms such as cell-penetrating peptide [28,29,30]. Therefore, in our study, although knocking out *CMAS* could significantly reduce the virus titer, IAVs were still replicating.

Studies have shown that the use of siRNA to silence the *ST3GAL4* gene could inhibit H5N1 avian IAV infection, and the gene was highly expressed in the human pharynx, trachea and bronchus, which consistent with our results [31]. The relationship between *ST3GAL4* and cancer occurrence had also been widely reported; for example, its expression was reduced in renal cancer cells and it was associated with the malignant development of subsequent cancers [32]. Other studies also have shown that the expression level of *ST3GAL4* in cervical cancer cells was also reduced [33,34]. Furthermore, there were also a few reports about the role of *ST3GAL4* in the replication of other viruses. The glycoprotein (GP) of the Lassa virus underwent conformational changes in the acidic environment of the late endosome and bond to the lysosomal transmembrane protein (LAMP1) to promote the fusion of the viral capsule and late endosome membrane, thus facilitating the replication of the Lassa virus [35]. In recent years, it has been found that *ST3GAL4* was necessary for the interaction of GP and LAMP1, and the interaction could not be detected in cells lacking *ST3GAL4* [36]. Furthermore, research evidenced that knocking out *ST3GAL4* had the least effect on cell growth and cell morphology compared with genes in the same family [37]. This study and our previous study on cell viability could prove that *ST3GAL4* was significant as a host factor against IAV.

In summary, our data suggested that knocking out *CMAS* and *ST3GAL4* hindered the synthesis of sialic acid receptors and resulted in the failure of the IAV HA binding to sialic acid receptors on the cell surface, which prevented the IAV from entering the cell for replication. The ultimate expectation of our research project is to create gene-edited animals that can resist influenza and facilitate the development of animal husbandry. These findings indicate that *CMAS* and *ST3GAL4* are very promising as potential targets for the production of gene-edited animals.

## 4. Materials and Methods

### 4.1. Cells and Viruses

Madin–Darby canine kidney cells (MDCK) were purchased from ATCC (Manassas, VA, USA). Human embryonic kidney 293T cells (HEK293T) and Newborn pig tracheal epithelial cells stably expressing Cas9 protein (NPTR-Cas9) were preserved in our laboratory. All cells were maintained with 5% CO2. IAVs used in experiments were A/swine/Hubei/221/2016 (Sw/HuB/H1N1), A/chicken/Shanghai/SC197/2013 (H9N2), A/swine/Henan/F26/2017 (Sw/HeN/H1N1), A/chick/Guangxi/YL01/2017 (H7N9) and A/Puerto Rico/8-SV14/1934 (PR8). All viruses were preserved in our laboratory, and experiments involving the H7N9 strain were carried out in the P3 laboratory.

### 4.2. Antibodies and Reagents

The antibodies used in experiments were anti-GAPDH and anti-HA mouse monoclonal antibodies (catalog no. PMK043F and PMK013C; PMK Bio, Wuhan, China); anti-Flag mouse monoclonal antibodies (catalog no. F1804; Sigma, Saint Louis, MO, USA); anti-IAV NP, M1 and HA rabbit polyclonal antibodies (catalog no. GTX125989, GTX125928 and GTX127357; GeneTex, Irvine, CA, USA); anti-*CMAS* rabbit polyclonal antibody (catalog no. WG-04641; ABclonal, Wuhan, China); Alexa Fluor 594-conjugated AffiniPure goat anti-rabbit and Alexa Fluor 488-conjugated AffiniPure goat anti-mouse secondary antibodies (catalog no. GR200G-43C and GM200G-02C; Sungene Biotech, Tianjin, China). The reagents used in experiments were DAPI (4′,6-diamidino-2-phenylindole; 1:1000) (catalog no. C1002; Beyotime, Shanghai, China); Biotinylated Sambucus Nigra (SNA) and Maackia Amurensis Lectin II (MAL II) lectins (catalog no. B-1305-2 and B-1265-1; Vector Lab, Burlingame, CA, USA) and Cy5-Streptavidin (catalog no. SA-1500-1; Vector Lab, Burlingame, CA, USA). We also used Protein A/G Magnetic Beads (catalog no. HY-K0202; MCE, Shanghai, China) and anti-HA immunomagnetic beads (catalog no. B26202; Bimake, Houston, TX, USA).

### 4.3. Plasmids

The *CMAS* gene was cloned into vector pCAGGS-HA and digested by Kpn I and EcoR I. The *ST3GAL4* gene was cloned into vector pCAGGS-HA and digested by Cla I and Nhe I. psPAX2 and PMD2.G were kindly offered by Professor Rui Luo (Hubei, China). LentiGuide-Puro was donated by Professor Shengsong Xie (Hubei, China). sgRNAs were designed according to the dedicated website (https://crispr.cos.uni-heidelberg.de/index.html?tdsourcetag=s_pctim_aiomsg, accessed on 20 April 2019), and were cloned into the LentiGuide-Puro vector to obtain *CMAS*-sgRNA and *ST3GAL4*-sgRNA plasmid to create lentivirus [38]. All primers are listed in Table 1.

### 4.4. Generation of the KO NPTr Cells

The single-guide RNA (sgRNA) sequence targeting the swine *CMAS* gene (5′- CACCGAGAACATTAAGCACCTGGCGGGG -3′) or *ST3GAL4* gene (5′- CACCGTTCAGGGTAGAAGAGACGCATGG -3′) were cloned into LentiGuide-Puro vector to produce the recombined lentivirus. The NPTr-Cas9 cells were infected with the *CMAS* or *ST3GAL4* LentiGuide-Puro lentivirus, and puromycin (2.5 μg/mL) was added to select the positive cells at 24 h post-infection (hpi) [39]. Then, the knockout efficiency of sgRNA was confirmed by sequencing at the genome level. We used the serially diluted method to grow the selected monoclonal cells.

### 4.5. Transfection

The plasmids and Lipofectamine 8000 (Invitrogen, Carlsbad, CA, USA) were mixed into Opti-MEM in proportion, and then the above-mixed solution was used to maintain 293T or NPTr cells. At 6 h post-transfection, the medium was replaced with a fresh medium.

### 4.6. Virus Titration

For NPTr cells in a 12-well plate, the supernatants were collected at designated time points after infection with IAV. MDCK cells were seeded into a 96-well plate, and the above supernatants were serially diluted in DMEM (Sigma, Saint Louis, MO, USA), and then added to each well in eight replicates of each dilution. After adsorption for 1 h, the inoculum was discarded, and the cells were washed with phosphate-buffered saline (PBS) and maintained with fresh DMEM. The MDCK cells were incubated at 37 °C for 72 h, and the virus titers were finally determined by calculating the 50% tissue culture infective dose (TCID50) using the Spearman–Karber method [40].

### 4.7. Western Blot Analysis

Correspondingly treated cells were lysed in a mannalian protein extraction reagent (Cowin Bio, Beijing, China) on ice, and then the cell lysates were added to 1 × SDS loading buffer for separation on SDS-PAGE, and then transferred to nitrocellulose (NC) blotting membrane for blocking for 1 h and then the corresponding antibody incubation. The images were obtained using a chemiluminescence imager (Tanon-5200, Shanghai, China).

### 4.8. Lectin and HA Binding Assays

Control and KO cells were fixed with 4% formaldehyde for 10 min, washed with PBS twice and incubated with 20 µg/mL biotinylated lectin for 1 h on ice, followed by staining with 1 µg/mL Cy5-Streptavidin. The levels of lectin binding were visualized using a confocal microscope (LSM 880; Zeiss, Oberkochen, Germany), or analyzed by a flow cytometer (Cytoflex LX; Beckman Coulter, Brea, CA, USA). Control and KO cells were infected with IAV for 60 min on ice, which allowed attachment but prevented internalization, then fixed. The cells were incubated with the corresponding primary antibody and fluorescent secondary antibody for 2 h and 1 h, respectively, and then the nuclei were stained with DAPI for 15 min at room temperature. A confocal microscope (LSM 880; Zeiss, Oberkochen, Germany) was used to obtain images.

### 4.9. Cell Viability Assay

The colorimetric-based cell counting kit-8 (CCK-8) assay (Dojindo Molecular Technologies, Rockville, MD, USA) was used to determine cell viability [39]. In short, control and KO cells were seeded in 96-well plates, and then 10 μL of CCK-8 reagent was added to each well at 12, 24 and 36 h. The plates were incubated in a 37 °C constant temperature incubator for 4 h and the absorbance at 450 nm was measured with a microplate reader [39].

### 4.10. Statistical Analysis

Data were shown as means ± standard deviation (SD) from three independent experiments. Statistical analysis was determined using a paired two-tailed Student’s *t* test or one-way ANOVA (* *p* < 0.05; ** *p* < 0.01; *** *p* < 0.001).

## Figures and Tables

**Table 1:** Primers used for PCR.

Primer Name	Sequences (5′ to 3′)
sgRNA Primers	
*ST3GAL4*-sgRNA	F: CACCGTTCAGGGTAGAAGAGACGCATGG
	R: AAACCCATGCGTCTCTTCTACCCTGAAC
*CMAS*-sgRNA	F: CACCGAGAACATTAAGCACCTGGCGGGG
	R: AAACCCCCGCCAGGTGCTTAATGTTCTC
PCR primers to verify mutations	
*ST3GAL4*-YZ	F: CGCCTCCCAGGCTAAACAAT
	R: GACACAGATCTCAAGTGTTCT
*CMAS*-YZ	F: TGGGAGGAAGATGGACTCAGT
	R: GCCTCCTTTCCTTTACCCAAT
PCR primers for gene cloning	
*ST3GAL4*-HA	F: CCATCGATATGGAGGAGGCTGGCCAGAG
	R: CTAGCTAGCTCAGAAGTATGTGAGGTTCTT
*CMAS*-HA	F: CCGGAATTCATGCCCCACAAGGGTGAA
	R: GGGGTACCTTAATTAATAGAAGGGCATGTCTTA

## Data Availability

Data is contained within the article or Supplementary Materials.

## Supplementary Materials

The following are available online at https://www.mdpi.com/article/10.3390/ijms22116081/s1, Figure S1: Validation of CMAS and ST3GAL4 knockout in NPTr cells, Figure S2: Determination of virus titer.
