# Supplementary material for: CMAS and ST3GAL4 Play an Important Role in the Adsorption of Influenza Virus by Affecting the Synthesis of Sialic Acid Receptors
Source: Int J Mol Sci. 2021 Jun 4;22(11):6081. doi: 10.3390/ijms22116081 (PMC8200212; doi:10.3390/ijms22116081)
Supplement: Supplementary file 1 [file ijms-22-06081-s001.zip › ijms-1229463-supplementary.pdf]

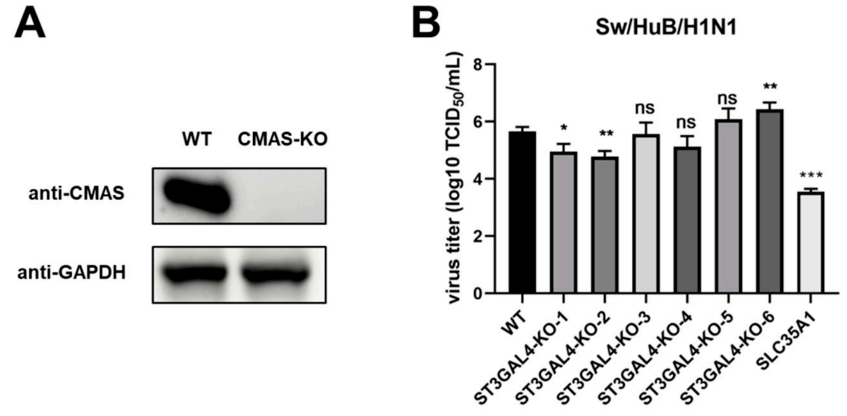

Figure S1. Validation of *CMAS* and *ST3GAL4* knockout in NPTr cells. (**A**) Detection of *CMAS* knockout efficiency by western blotting. WT NPTr cells and *CMAS*-KO NPTr cells were collected and lysed for western blot assay using the rabbit anti-*CMAS* antibody and the anti-GAPDH antibody. GAPDH was used as the loading control. (**B**) WT NPTr cells and *ST3GAL4*-KO NPTr cell lines were infected with the Sw/HuB/H1N1 strain, and the supernatants were collected at 36 hpi to detect the virus titer (Data from three independent experiments, mean ± SD; *, $P < 0.05$; **, $P < 0.01$; ***, $P < 0.001$; ns represented no significance; two-tailed Student's t test).

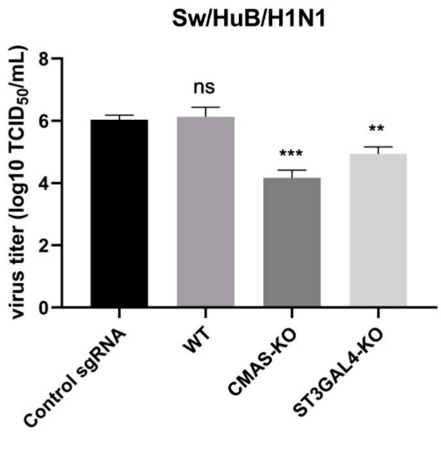

Figure S2. Determination of virus titer. NPTr cells infected with control sgRNA lentivirus, Cas9 expressing NPTr cells and KO cells were in-fected with the Sw/HuB/H1N1 strain, and the supernatants were col-lected at 36 hpi to detect the virus titer (Data from three independent experiments, mean ± SD; **, $P < 0.01$; ***, $P < 0.001$; ns represented no significance; two-tailed Student's t test).
